# The Arabic version of the original (long-form) neurogenic bladder symptoms score validity and reliability in patients with lower urinary tract disorders: A multi-center evaluation

**DOI:** 10.1080/20905998.2025.2555083

**Published:** 2025-09-01

**Authors:** Diaa-Eldin Taha, Ali Ibrahim, Abdelwahab Hashim, Salem Bahdilh, Ibrahim Alowidah, Mohamed Ehab, Ali Abdel Raheem

**Affiliations:** a Urology Department, Kafrelsheikh University, Kafrelsheikh, Egypt; b Urology Department, King Saud Medical City, Riyadh, Saudi Arabia; c Urology Department, Faculty of Medicine, Delta University for Science and Technology, Dakahlia, Egypt; d Urology Department, College of Medicine, Tanta University, Tanta, Egypt

**Keywords:** Neurogenic bladder symptoms score (NBSS)

## Abstract

**Background:**

The long (original) form of neurogenic bladder symptoms score (NBSS) entails full assessment of the neurogenic status of the patient that is not included in the short form. The aim of this study was to examine the psychometric qualities of the Arabic NBSS among those suffering from neurogenic lower urinary tract dysfunction (NLUTS).

**Methods:**

The Arabic-language study included patients with NLUTS. Among the psychometric attributes that were looked at were construct validity, content validity, internal consistency, and test–retest dependability. Cronbach’s alpha and the Intraclass Correlation Coefficient (ICC) were used, respectively, to assess internal consistency and test–retest reliability. In order to evaluate construct validity, the Qualiveen questionnaire was compared to the NBSS.

**Results:**

The study included 255 NLUTS patients. The internal consistency of the NBSS overall score was found to be variable, with Cronbach’s α of 0.82 for both the overall score and each of its subdomains (urinary incontinence: 0.94, storage/voiding: 0.86, consequences: 0.88). Thirty days (±3.6 days) after the baseline version, 233/247 (84%) patients completed and returned their questionnaires for test–retest reliability. The overall score had an ICC of 0.92, while the subdomains of storage/voiding, urine incontinence, and repercussions had ICCs of 0.92, 0.83, and 0.80, respectively. The correlation study demonstrated the strong construct validity of the Arabic NBSS-SF version.

**Conclusion:**

Relevant psychometric characteristics, including content, construct test–retest reliability, internal consistency, and validity, can be found in the Arabic version of the NBSS. The NBSS in Arabic is appropriate for usage in study and clinical contexts.

**Clinical trial registration No:**

H1R1-27-Dec22-01-MKSU 51-1-16.

## Introduction

The malfunction of the neurogenic bladder can be caused by any disease or injury that affects the neurological conditions, like spina bifida, multiple sclerosis, or spinal cord injuries [1]. Upper urinary tract problems, such as urinary tract infections (UTI) or renal failure, may be caused by neurogenic bladder [2], its symptoms have been connected with a poor quality of life (QOL) among SCI [3] MS [4] and SB [5] patients, and have an important socio-economic impact [6].

Those having symptoms related to the lower urinary tract or bladder dysfunction account for more than 80% of cases of multiple sclerosis (MS) and SCI [6,7]. Because of their neurogenic bladder, these patients need particular care, which may need an indwelling catheter or intermittent bladder catheterization on a periodic basis. All current medications and treatments aim to improve the quality of life for patients suffering with neurologic bladder disorders [7,8].

There are various instruments or questionnaires available to investigate LUTS. Recently, novel techniques for evaluating symptoms, life satisfaction, and quality of life have been established and proven. These instruments, which correlate to patient-reported outcomes (PROs), enable the assessment of patients’ perspectives directly without the need for clinical judgment. These evaluation instruments were first created in oncology but are now more often used in general urology. In fact, PROs are suitable for evaluating symptoms when there are no risk factors for the condition, such as overactive bladder or urine incontinence, and could help with therapeutic therapy [9].

There isn’t a single metric that can be applied to assess NLUTS in every clinical research study. Because of their robust psychometrics and widespread application in SCI, it is advised to use Qualiveen and the Neurogenic Bowel Dysfunction Score (NBDS) as supplementary, highly recommended measures [10].

In 2013, Welk et al. developed the NBSS [11]. A psychometric multidimensional tool called the NBSS is used to evaluate NLUTS in patients suffering from neurological conditions. The first of its 24 questions characterizes people who utilize medications for bladder management. The final question assesses the overall quality of life. Three domains are examined in the next 22 questions: consequences, incontinence, and voiding [11,12]. Then, in 2020, Welk et al. created the NBSS-SF, a shortened edition of the NBSS that had 10 questions and evaluated the three domains that were similar to the NBSS original version [13].

In addition to being assessed in individuals with MS [14], SCI [15], and adults who encounter cerebral palsy [16]. Several languages have been used to validate the NBSS, such as Russian [17], Turkish [18], Portuguese [19], and Greek. Additionally, the NBSS-SF has been validated in French [20] and Arabic language [21].

The Arabic language is extensively spoken, and many patients suffer from NLUTS. Our aim is to validate the original long form of NBSS that precludes many items in patient assessment that are not included in the short form.

## Materials and methods

The Ethics Committees have given their approval to this project. Institutional Review Board Letter of Consent. Every patient gave their informed consent and willingly consented to participate in the study in line with the Helsinki Declaration.

### Cross-cultural adaptation, both forward and backward translation

The criteria offered by Beaton et al. for self-reported metrics served as the basis for translating the NBSS questionnaire [22]. The instrument’s modification was made possible by the inventor of NBSS. Two interpreters, each with a native Arabic language, carried out separate forward translations to convert the English source version of the questionnaire to its Arabic target language. After that, the multilingual translators convened to assess each translated word, term, and sentence in the questionnaire. Based on their collective assessment, the first Arabic version was produced. After that, a natural English speaker with strong Arabic language skills but had no preceding medical training translated the first Arabic version back to English without realizing the intention of the study. The authors then went over the English back translation and looked for any differences with the Arabic original. A multilingual committee made up of one specialist in bladder neurologic abnormalities (SB) and two neurorehabilitation professionals (DT, AA) reviewed the results. The items of the instrument were analyzed to ascertain each version item’s semantic, idiomatic, and conceptual equivalency, as well as its clinical significance and appropriateness for application in the specific patient population [22]. The modifications recommended were only adopted when there was approximately 90% agreement [22]. To determine whether alternative responses were understandable along with how successfully the 15 Arabic patients with NLUTS understood the questionnaire items, the last stage involved a pilot study employing the NBSS’s Arabic version (7 women and 8 men). Based on the comments from 15 participants, the committee made a few minor changes. Several adjustments later, the last version in Arabic was completed.

### Acquisition of patients

This study recruited 255 participants from two Arabic centers, one in Egypt and the other in Saudi Arabia, between December 2021 and January 2025. Participants had to be at least 18 years old, have a diagnosis of spinal cord injury or multiple sclerosis (MS), and be fluent in Arabic. The criteria for exclusion were (i) those who received performed urologic surgery within the last year, (ii) an upsurge in overall health, and (iii) a shift in the signs and symptoms they experienced while receiving treatment for a continuing NB or infection in the urinary tract within the last 30 days.

### Questionnaire

A self-reported tool entitled the NBSS questionnaire was created to analyze lower urinary tract symptoms as well as the effects of NB. The NBSS questionnaire encompasses 25 measures that are specific to urine symptoms alongside eight neurogenic qualities of life metrics. The most common themes included urgency, bladder spasms, urine incontinence, and urinary tract infections. The 25-item scale evaluates three areas of quality of life (QOL): urination (three items), storage (five items), and incontinence (seven things), QOL (two questions), and repercussions (seven items) [8]. To evaluate test–retest reliability, all participants were asked to refill the questionnaire within 20–30 days. The interval that existed between each test was just sufficient to lessen the possibility that the patients will misunderstand the questions, but also quick enough to stop any alterations in their bladder management techniques or symptoms.

### Psychometric properties

The psychometric parameters of the questionnaire for the NBSS have been investigated in our study utilizing the cross-cultural adjusting process criteria [23]. Reliability is the degree to which a measurement is error-free [24]. This study looked at measurement features, internal consistency, and test–retest reliability. The degree of congruence between items in the same subscale or domain is referred to as internal consistency. Utilizing Cronbach’s alpha, it is measured. More than 0.70 on the internal consistency scale is considered adequate [25].

Using the NBSS scores from visits 1 and 2, an assessment was conducted on the NBSS questionnaire’s test–retest reliability. Accordingly and since we employed the ICC (intraclass correlation coefficient) for the test-retest reliability study, we declared intraclass correlation coefficients for reliability research using the two-way mixed effect model [26]. Strong correlations are those with a value of more than 0.7 [25].

The degree to which a measure’s results accurately reflect what it is intended to measure is referred to as validity [24]. We assessed the content and construct measurement attributes in our work.

The capacity of an instrument to quantify the fundamental idea it aims to identify is termed as construct validity [25]. Using a comparison of the Arabic validated Qualiveen questionnaire [27] and Spearman’s rank correlation, the construct validity of this study has been assessed.

Professionals in the area can determine if the scale’s inquiries are thorough and pertinent to the issue that needs to be assessed. Content validity accounts for the extent which an instrument is attached for evaluation is representative of and relevant to the intended to build it is intended to measure [25]. Two physicians (DT, AA), one of whom experienced in neurologic disorders of the bladder and the other in urogynecology, reviewed the content validity of this study. They were both part of the expert panel that was responsible for translating the NBSS into Arabic.

The estimations for the sample proportions were based on a minimum of 10 patients are needed for each question, as advised in the literature [28]. We had 255 patients. Consent in writing was acquired. The ethics committee gave the study its approval.

### Statistical analysis

When dealing with continuous data, the mean and standard deviation are used. Baseline attributes are provided as percentages when working with categorical data. To evaluate the NBSS’s internal consistency, Cronbach’s alpha was employed. (Less than 0.50 = undesirable; 0.6–0.5 = poor; 0.7–0.6 = dubious; 0.8–0.7 = acceptable; 0.9–0.8 = outstanding; greater than 0.90 = exceptional) was considered to be a value of 0.7 or above. SPSS Version 23.0 was used for all statistical analyses, with a fixed significance level of 0.05.

## Results

The study included 255 patients, 128 of whom were men (49.3%). The mean age (±SD) was 39.8 (±13.2). Table 1 displays other demographic data, clinical features, neurological disease duration, damage severity, level of education, and bladder treatment method of the individuals.

**Table 1.** Demographic and clinical characteristics of patients.

Variable		Value
Gender	Male	128 (51%)
	Female	127 (49%)
Age (mean ± SD)		39.8 (±13.2)
BMI (mean ± SD)		26.92 ± 5.89
Marital status	Single	158 (56%)
	Married	97 (44%)
Education level	High school	57 (17%)
	University	198 (83%)
Aetiology	MS	110 (39.2%)
	SCI	145 (60.8%)
Neurogenic level	Cervical	41 (10.3%)
	Thoracic	32 (9.8%)
	Lumbar/sacral	72 (79.9%)
Voiding methods	CIC	59 (36.3%)
	Indwelling catheter	26 (8.6%)
	Spontaneous	170 (55.1%)
Duration of LUTs (months) (median, range)		6 (6–30)
LUTs types	Storage	197 (74.4%)
	Voiding	20 (9.6%)
	Storage and voiding	38 (16.0%)

Out of the 255 individuals who replied to the baseline NBSS questionnaire, 12 (9.9%) opted not to retake it 30 days (±3.6 days) afterwards. Data from 255 participants who finished both the test and retest phases have been utilized for the test–retest reliability analysis. A 42.04 ± 6.75 was the average overall Qualiveen score, while 56.22 ± 5.03 was the average overall NBSS score.

The results of analyzing each of the NBSS subdomains independently were 0.57 for the consequences domain, 0.72 for storage and voiding, and 0.84 for incontinence (Table 2). The Arabic NBSS, on the other hand, had an overall Cronbach’s alpha score of 0.82 when all items, including the QOL question, underwent scrutiny. In the test–retest reliability study, an ICC of 0.91 (95% CI 0.90–0.94, *p* < 0.001) was found for the total score and for each subdomain separately (ICC of 0.94 for incontinence, 0.72 for storage and voiding, and 0.90 for consequences) (Table 3).

**Table 2.** Correlation between Qualiveen and NBSS scores.

Neurogenic bladder symptom score	SCI	MS	P
Incontinence domain (mean ± SD)	15.22 ± 8.16	11.95 ± 8.41	0.03
Storage and voiding domain (mean ± SD)	11.47 ± 4.43	10.65 ± 4.64	0.32
Consequences domain (mean ± SD)	7.50 ± 3.60	7.08 ± 3.69	0.53
QOL domain (mean ± SD)	3.28 ± 2.48	3.12 ± 2.21	0.70
King’s Health Questionnaire General health perception (mean ± SD)			
Incontinence impact (mean ± SD)	39.63 ± 24.42	29.12 ± 23.53	0.12
Role limitations (mean ± SD)	36.24 ± 21.91	33.25 ± 22.74	0.02
Physical limitations (mean ± SD)	38.3 ± 22.7	41.1 ± 26.5	0.84
Social limitations (mean ± SD)	50.4 ± 24.1	54.5 ± 24.4	0.001
Personal relationships (mean ± SD)	50.6 ± 30.5	68.6 ± 27.9	0.07
Emotions (mean ± SD)	49.8 ± 28.7	32.1 ± 23.1	0.97
Sleep/energy (mean ± SD)	56.9 ± 24.9	57.7 ± 27.4	0.01
Severity measures (mean ± SD)	41.1 ± 26.5	38.4 ± 26.1	0.07

**Table 3.** The reliability of the Arabic version of the neurogenic bladder symptom score.

	Internal consistency		Test–retest analysis	
	Number of items	Cronbach’s alpha	ICC value	p
Incontinence domain	8	0.94	0.92	0.02
Storage and voiding domain	7	0.86	0.83	0.00
Consequence domain	7	0.88	0.83	0.04
Total	23	0.79	0.80	0.01

The construct validity was evaluated using the Spearman’s rank test. The Qualiveen and NBSS showed a strong positive connection (*r* = 0.73, *p* < 0.001). Likewise, item 2 of the NBSS showed notable moderate negative associations with the SF-12 subdomains of physical and mental health (*r* = −0.42, *p* = 0.001, and *r* = −0.61, *p* = 0.003, respectively).

## Discussion

In order to ensure the effectiveness of assessment tools created in a foreign language while taking linguistic and cultural differences into consideration, a methodical and regulated process of cultural adjustment and validation is necessary [22]. This method opens the door to multicenter trials including other countries and permits the establishment of standards and comparisons with global data [29].

In general, the psychometric properties of the Arabic version of the NBSS are similar to those of the English version. Cross-cultural adjustment produces positive acceptance and comprehension outcomes. With the exception of three minor flaws, the methodological technique produced no problems during translation or back-translation by independent translators. Specifically, the translations of ‘urgency,’ ‘bladder reservoir,’ and ‘penile condom’ did not match the correct Arabic phrasing.

The majority of patients completed the questionnaire with ease, while a few needed topics clarified. We think this is because we followed a thorough and exacting process when interpreting foreign standards [22]. Owing to simple use, even patients living in rural areas Without access to formal education, individuals might use the self-applicable NBSS to figure out how their illness is progressing.

The NBSS version’s psychometric properties, including test–retest reliability, construct and content validity, and internal consistency, have been proven in the current analysis. Similar to the first validation trials (Cronbach’s alpha = 0.76), the version of the NBSS in Arabic demonstrated internal consistency for the overall score (Cronbach’s alpha = 0.82) [8], and the French version (Cronbach’s alpha = 0.79) [20]. The three subdomains’ Cronbach’s alpha values were consequences (0.88), storage/voiding (0.86), and incontinence (0.94). For the French version, S. Berradja et al. showed a comparable pattern (0.86, 0.71, and 0.43 in that order) [20], and by B. Welk and associates for the first iteration (0.91, 0.69, and 0.25, correspondingly) [8].

Low internal consistency was found in the consequences domain, as stated by Welk et al. [8]. Our study sample’s low internal consistency in the consequences domain and quality of life subscales may be attributed to the large proportion of patients receiving CIC treatment. The fact that over 50% of our population relied on CIC for urinary control may help to explain this finding.

We found an ICC of 0.92 regarding the dependability of tests after a median of 15 days, which is consistent to the validation research executed among French patients (0.90) [20] significantly higher than the ICC value (0.84) from the initial research [8]. By comparing Question 2 of the NBSS to the Arabic versions of Qualiveen, the QoL questionnaire, we were able to ascertain the construct validity. Comparing our study to the Qualiveen-SF HRQOL questionnaire, we found an important correlation (*r* = 0.73) with the Qualiveen, which was higher than the original English version’s outcome (*r* = 0.67) [8]. The association with the 12 subdomains of physical and mental health (*r* = −0.46) and mental health (*r* = −0.55) in SCI patients was similar to a prior study using the original form [15].

The study contains a lot of shortcomings. First, there is disagreement over the ideal sample for cross-cultural adaptation and validation research, despite the fact that it is vital for study design. Van Nispen and associates [30] argued that as the problem is heavily dependent on the construct to be evaluated and that the present proposals do not fully describe a suitable sample size, researchers should decide what the proper sample size is for their investigations. Terwe et al. [31] observed that proposals for the appropriate sample size in measurement-based research are not clearly stated. Herein, we take 255 patients as a sample size. Second, we couldn’t find a long questionnaire to construct validate the current long form, so we depended on the short form of Qualiveen (Qualiveen-SF). Third, the validation is done in two Arabic countries with different cultural backgrounds, but the questionnaire is already in the written formal Arabic language that is well known to all different literate Arabic cultures.

We were able to validate and cross-culturally modify the NBSS questionnaire for the Arabic community in spite of the limited sample size. Our work satisfies the fundamental requirements set out by Terwee et al. [31] and is similar to other validation studies [32,33].

## Conclusion

Relevant psychometric characteristics, including internal consistency, test–retest reliability, content, and construct validity, can be found in the version of the NBSS in Arabic. It is a handy and trustworthy tool for evaluating NB-related quality of life in the Arabic-speaking community of individuals with NLUTS.

## Data Availability

The datasets used and/or analyzed during the current study are available from the corresponding author on reasonable request.
